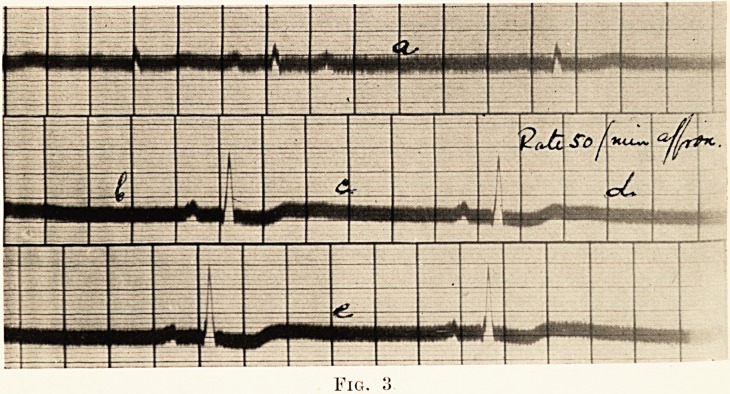# Three Cases of Sino-Auricular Block

**Published:** 1929

**Authors:** H. L. Heimann

**Affiliations:** Extra Honorary Physician, Johannesburg General Hospital


### THREE CASES OF SINO-AURICULAR BLOCK.

BY

H. L. Heimann, M.D., M.R.C.P.,

*Extra Honorary Physician, Johannesburg General Hospital.*

---

*Physiology.* — Sinus arrhythmia is a well - known physiological condition in which there is rhythmical variation in the heart-rate due to vagal influences at the pace-maker. The variation may be dependent on the respiration, the rate waxing with inspiration, waning with expiration, or it may be unrelated to breathing. The condition is well marked in children and athletes of great stamina. It is common in the latter to find a pulse-rate of 44 to 50 per minute when in training, and for this rate to double itself abruptly on exercise when vagal influences become lessened. The doubling is too sudden to be ascribed to chemical changes in the blood, as this produces a gradual increase of pulse-rate.

If we consider a rhythm in which vagal influences cause a sinus arrhythmia, we can see that if this influence becomes accentuated at the appropriate point, it is possible that it will prevent the formation of one of the rhythmical auricular impulses at the sinus venosus. As no excitatory state will pass down the bundle of His no ventricular contraction will occur. There follows a gap in the heart cycles of a complete auricular and ventricular beat, and this is called sino-auricular block. It is represented on the radial curve by a missed beat indistinguishable from a missed beat of true auriculo-ventricular block, on the venous curve by the absence of the a—c—v waves for one cycle, and on the electro-cardiograph (the most certain method of detection) by the absence of P Q R S and T waves for one cycle. It follows that both heart-sounds are absent for this cycle, but this alone does not distinguish the phenomenon from auriculo-ventricular block.

The condition, if often repeated, causes giddiness and a feeling of palpitation due to the irregularity. In my experience it is more common in this country than in England, and I have already described two cases.[1] In neither of these was I fortunate enough to obtain a picture of the actual block, but the tracings showed marked vagotonia, and the history suggested this condition. The three cases described below were definitely proved to be examples of sino-auricular block by tracings exhibiting the characteristic pause.

*Ætiology.*—In my previous article it was suggested that the condition might be due to suprarenal exhaustion due to the combination of hard work with the effect of excessive sunlight. The latter factor has not been noted in these present cases, two of whom were medical practitioners and one a typist. It is possible that there was suprarenal exhaustion due to other causes, and hence a vagotonia following on lack of sympathetic tone.

The condition is not pathological in itself. Unless it accompanies true auriculo-ventricular block or other organic mischief, the prognosis is excellent, and the patient should be considered from the aspect of vagotonia alone.

**Case 1.**—This patient was a man aged 59. He complained of periodic attacks of palpitation of irregular type, beginning and ending suddenly. This was accompanied by an indefinite

Fig. 1.

Fig. 2.

Fig. 3

abdominal discomfort, relieved by going to stool. He was losing weight and vigour, and his sphere of activity was limited. The history dated from the strain of a motor touring race some five years previously. His pulse-rate was persisting at a rate from 80 to 100 per minute—much higher than five years previously, when it had been on the low side.

On examination there was found no enlargement of the heart and no valvular defect. The heart and pulse became totally irregular during the attacks, which were proved by the electrocardiograph to be due to auricular fibrillation. Between the attacks the pulse showed irregularities occurring at intervals, and, on listening, spaces were heard in the normal rhythm. The tracing shown here proved these to be due to sino-auricular block. (Fig. 1.)

The thyroid gland was found to contain a small adenoma. The clinical picture was completed by slight exophthalmos and find tremor of the hands. A diagnosis of toxic adenoma of the thyroid was made. This was removed by Mr. Daly, under local anæsthesia given by Mr. Levin. Some septic teeth were also removed.

The patient made a good recovery. He has had no recurrence of the attacks of palpitation, and the electrocardiograph shows no evidence of sino-auricular block. He has gained in weight and vigour, and his pulse-rate ranges around 72 per minute.

**Case 2.**—F., aged 35. She complained of periodic attacks of faintness and giddiness, following chloroform anæsthesia for the reduction of piles and prolapse of the rectum.

The pulse was irregular, and the systolic blood-pressure only 95 millimetres of mercury. The pulse slowed considerably, and became more irregular when the breath was held. There was no change in the size of the heart from normal, and no thrills or murmurs.

The electro-cardiograph showed sinus block at *a* and *b*. (Fig 2.) Belladonna in five mimim doses of the tincture three times a day relieved her considerably. Some septic teeth were also removed. She made a good recovery.

**Case 3.**—F., aged 20. This patient complained of attacks of palpitation, shortness of breath and pain on the left side of the chest, sharp in character, accompanying the palpitation. The attacks had been present for five years, and they began and ended suddenly. She was not seen in an attack, so its nature could not be identified. She had scarlet fever when 10 years old, chorea when 14 years old.

On examination the heart was found to be normal in size, and there were no thrills or murmurs. An irregularity was present, which became more marked on holding the breath. This irregularity was identified by the cardiograph as sino-auricular block at *a*, *b*, *c*, *d*, *e*. (Fig. 3.)

It is not clear whether paroxysmal tachycardia of one or another variety occurred. I have seen no evidence of it. There was no evidence of thyroid toxicity as in Case 1.

REFERENCE.

[1] *S. African Med. Journ.*, September, 1929.

## Figures and Tables

**Fig. 1. f1:**
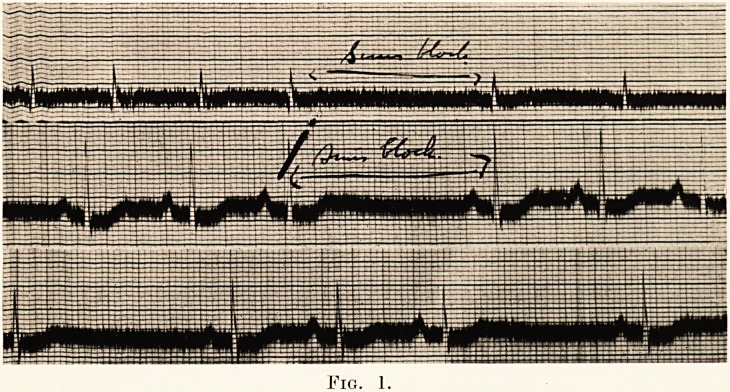


**Fig. 2. f2:**
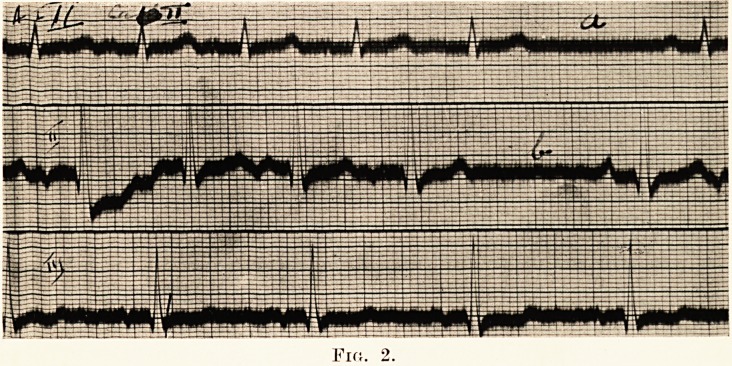


**Fig. 3. f3:**